# How do we strengthen the health workforce in a rapidly developing high-income country? A case study of Abu Dhabi's health system in the United Arab Emirates

**DOI:** 10.1186/s12960-019-0345-9

**Published:** 2019-01-24

**Authors:** Marília Silva Paulo, Tom Loney, Luís Velez Lapão

**Affiliations:** 1 Institute of Public Health, College of Medicine and Health Sciences, United Arab Emirates University, PO Box 17666, Al Ain, United Arab Emirates; 2 Global Health and Tropical Medicine, Instituto de Higiene e Medicina Tropical, Universidade Nova de Lisboa, Lisbon, Portugal; 3 College of Medicine, Mohammed Bin Rashid University of Medicine and Health Sciences, Dubai, United Arab Emirates

**Keywords:** Abu Dhabi, Chronic Care Model, Health workforce, Health systems, United Arab Emirates

## Abstract

**Background:**

The United Arab Emirates (UAE) is a rapidly developing high-income country that was formed from the union of seven emirates in 1971. The UAE has experienced unprecedented population growth coupled with increased rates of chronic diseases over the past few decades. Healthcare workers are the core foundation of the health system, especially for chronic care conditions, and the UAE health workforce needs to be fully prepared for the increased rates of chronic diseases in the adult population. Abu Dhabi is the largest emirate in terms of land mass and population size, and the purpose of this paper was to assess how the health system has been using the Chronic Care Model to improve its capacity to reach out to all patients in the population.

**Case presentation:**

The Abu Dhabi health workforce has twice the number of doctors (52.4 vs. 23.2 per 10 000 population) and nurses (134.7 vs. 50.4 per 10 000 population) compared to the entire UAE health workforce. In addition to an overreliance on expatriate workers, there is an excess of some specializations such as general medicine and gynecology and a severe undersupply of other specialties including trauma and injury, and medical oncology. The digital infrastructure and skills of the health workforce need to be improved to minimize the proportion of the appointment time required to complete administrative tasks for a health insurance system and maximize the doctor-patient face-to-face interaction time for consultation and lifestyle counseling.

**Conclusions:**

A greater emphasis needs to be placed on developing self-management support strategies using a combination of nurse health educators and community-based patient-led health programs. The UAE Vision 2021 includes developing a world-class healthcare system, and full implementation of the Chronic Care Model seems to facilitate the detailed planning and preparation of healthcare services and workers required to achieve this goal.

## Background

Strategies to achieve Universal Health Coverage and the Sustainable Development Goals have been the main themes for discussion and allocation of resources among healthcare policymakers. Both of these initiatives target the organization and associated costs of healthcare services required to reach the entire population of a specific country. Strategic planning and systematic resolutions coupled with sustained political commitment are needed to achieve long-term improvements in health at the population level. The World Health Organization (WHO) is reinforcing the importance of an effective, efficient, and adequate health workforce as one of the six key building blocks for any health system [1]. Recently, the *Global Strategy on Human Resources for Health: Workforce 2030* [2] was published to raise awareness about the importance of strengthening the health workforce to better address the new challenges of integrating people-centered health services. The WHO proposed a specific framework of action for health workforce development in the Eastern Mediterranean Region (EMR) [3]. This region is facing major challenges regarding the capacity and composition of the health workforce with an “overall shortage of qualified workers with suboptimal and imbalanced overall production and availability in the region” [3]. To address this challenge in the EMR, the WHO issued policy directions with the focus on the development and implementation of information-based strategic plans to optimize availability, quality, and performance; to strengthen governance and regulations; and to invest in the current and future needs [3]. The EMR countries have also been divided into three groups according to the specific needs of their health workforce. Group 3 includes Yemen, Pakistan, and Afghanistan which are countries that have critical shortages of, low production of, and poor performance of healthcare workers. Group 2 is composed of Egypt, Iran, Iraq, Palestine, and Tunisia that have challenges related to the limited employment capacity and an imbalance in the skill composition of the workforce. Group 1 includes Bahrain, Kuwait, Oman, Qatar, Saudi Arabia, and the United Arab Emirates (UAE). This group has major issues related to a shortage of national health workers, a high reliance on expatriate staff, limited professional production capacity, and a high turnover of expatriate staff [3].

The focus of this case study is the UAE which is located in the southeast of the EMR in the Arabian Peninsula. The UAE is considered a young but rapidly developing high-income country that resulted from the union of seven emirates in 1971: Abu Dhabi, Dubai, Ajman, Umm Al Quwain, Sharjah, Fujairah, and Ras Al Khaimah. The health systems within each of the seven emirates have distinct federal and emirate level authorities related to the regulation and provision of healthcare in that specific emirate. The federal level is regulated by the Ministry of Health and Prevention, and the emirate level is regulated by the different specific emirate entities such as the Department of Health Abu Dhabi, the Dubai Health Authority, the Dubai Healthcare City (a healthcare economic free zone), and the Sharjah Health Authority. Extensive health system reforms have been implemented across the seven emirates by the different health regulators over the past decade, and the UAE health system is currently internationally competitive and well ranked [4–6]. However, continued strategic developments are required to align the services and programs to best international practices [4, 5]. The UAE government has invested significant resources into developing the health system to achieve a World-Class Healthcare by 2021 - the UAE Vision [7]. The UAE Vision 2021 World-Class Healthcare Agenda defined national priority areas comprising 10 indicators including healthcare services (percentage of accredited healthcare facilities, healthcare quality index), human resources for health (number of physicians and nurses), and chronic diseases (prevalence of diabetes and obesity in children, average healthy life expectancy, percentage of smoking, and number of deaths from cardiovascular diseases and cancer) [8]. These indicators will also help the UAE to achieve the third Sustainable Development Goal which aims to “ensure healthy lives and promote well-being for all at all ages” [9].

Each emirate in the UAE has its own specific health strategy, and as the UAE has seven emirates with different health regulators, this paper will focus on Abu Dhabi emirate which is the largest in terms of land mass and population [10].

### The setting of the research

In mid-2016, the total population of the emirate was estimated to be 3 037 937 people, with a compound annual population growth rate of 4.6%, since 2011 [11]. The distribution of the population is unequal between the three regions of the emirate: Abu Dhabi Region, Al Ain Region, and Al Dhafra Region. From the total population, 53.3% live in the Abu Dhabi Region, 41.0% live in the Al Ain Region, and 5.7% live in the Al Dhafra Region. More than three quarters of the total population of the emirate, 81.9%, are expatriates, and 64.2% of those live in the Abu Dhabi Region [11]. This region hosts the city of Abu Dhabi, capital of the country and where the federal government is based. There is also a gender disproportion in the population of the emirate where 64.0% are males. This disproportion is similar to the situation of the other emirates, such as Dubai [12], and other Gulf Cooperation Council countries such as Qatar and Bahrain [13, 14]. The mass recruitment of males for industrial and construction sectors is the reason for this unusual population structure [10]. Chronic diseases are the majority of the diseases encountered in Abu Dhabi’s health system. In 2016, diseases of the circulatory system were responsible for 37.0%, neoplasms for 15.0%, and endocrine, nutritional, and metabolic diseases for 2.0% of deaths of the Abu Dhabi population [15]. In the emirate of Dubai, circulatory system diseases were responsible for 33.4%, neoplasms for 17.9%, and respiratory system diseases for 11.1% of deaths in the emirate [12]. Concerning the whole UAE, the Institute of Health Metrics and Evaluation ranks ischemic heart diseases, cerebrovascular diseases, chronic kidney disease, and diabetes as the four non-communicable diseases that cause most of the deaths in the UAE [16]. In addition, these four diseases along with road injuries and congenital defects are the top five causes of premature death in the country [16].

## Case presentation: Abu Dhabi healthcare system

The Abu Dhabi Healthcare Strategic Plan was launched in December 2014 by the Department of Health—Abu Dhabi. It is a comprehensive plan that translates the commitment of the government to improve the healthcare services, and it has 58 initiatives covering the seven priority areas: reducing capacity gaps; improving the quality of healthcare services, patient safety, and experience; attracting, training, and retaining qualified healthcare professionals; improving preparedness during emergencies; ensuring value for money and sustainability of healthcare spending; and introducing an e-health program as a facilitator for the other priorities [17]. A recent modified-Delphi study recruited a purposive sample of health systems’ experts to reach consensus on and rank the top five priorities and the top five barriers for the development of chronic care in Abu Dhabi [18]. The modified-Delphi study revealed the following priorities: (i) organizational leadership in chronic illness care, (ii) continuity of care and effective behavior change intervention and peer support, (iii) evidence-based guidelines, (iv) improvement strategy for chronic illness care, and (v) provider education for chronic illness care [18]. The top ranked barriers were (i) patient compliance, (ii) lack of standardized processes/procedures, (iii) differences between insurance coverage, (iv) a lack of regional plan standardizing guidelines between facilities, and (v) a lack of monitoring (e.g., patient-related outcomes and safety, hospital performance) [18].

The primary aim of this paper is to contribute to improving the capacity of healthcare workers to reach out to chronic patients through the identified gaps of the Chronic Care Model (CCM) in the primary healthcare system of Abu Dhabi. The CCM is a framework for improving chronic illness care at both the individual and population-level; it is an evidence-based guideline delineating the health system as part of the community [19]. The CCM focuses on population-based daily care for all with structured and planned team care interventions delivered through the integration of six elements: health system’s organization, community, self-management support, delivery system design, decision support, and clinical information systems [20]. The model is a framework to approach chronic diseases and it organizes the healthcare services, the use of technology, and family or patient independence and autonomy. The CCM has been used to both promote changes in the delivery of care for individuals living with chronic diseases that can lead to advances in health outcomes [21, 22] and as a tracking tool to identify areas for improvement in the organizational setting and delivery of care [23–26]. A recent systematic review of the Abu Dhabi’s health system identified specific gaps in the development and implementation of the CCM within the emirate [4]. The Abu Dhabi’s health system, through the Ambulatory Healthcare Services, implemented the Patient-Centered Medical Homes (PCMH) in 2013. The PCMH model is aligned with the CCM and was designed to incorporate the same components. For example, the PCMH model has the similar objective of providing structured, proactive, and coordinated care with the patient in the center surrounded by a primary care physician, a dietician, a nurse case manager, a counselor, a specialty physician, and key infrastructure and services such as laboratory, radiology, pharmacy, and the hospital [4]. Similar to the CCM, the PCMH team work is organized around the patient’s needs aiming to provide quality care.

## The use of the CCM as an approach for improving the patient-interaction of the health workforce

### Strengthening the health workforce in Abu Dhabi

The emirate of Abu Dhabi reports that in 2015 there were 41 882 health professionals registered, in both private and public sector healthcare facilities [27], and in 2016, the number increased to 49 007 health professionals [11]. In 2015, there were 52.4 physicians and 134.7 nurses per 10 000 population (adjusted for the young population) in Abu Dhabi emirate [27], compared to 22.3 physicians and 50.4 nurses per 10 000 population in the entire UAE population [28]. The Department of Health of Abu Dhabi estimates that by 2035 the number of physicians will need to increase by 50% in the emirate to cope with the projected population growth and rising rates of chronic diseases [11].

**Fig. 1:**
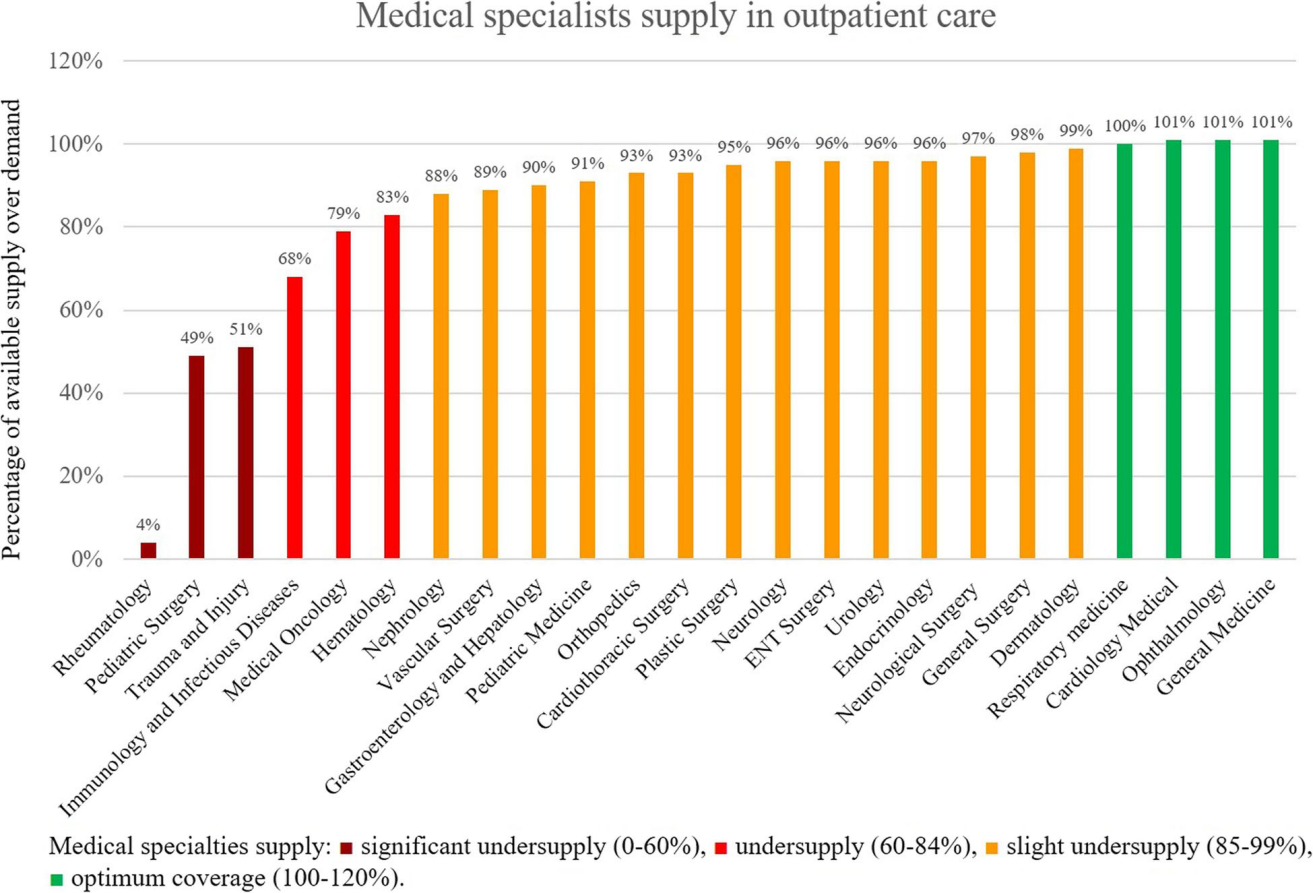
Percentage of medical specialists supply at the outpatient care (hospitals and primary care) in Abu Dhabi. Maroon indicates significant undersupply (0–60%), red indicates undersupply (60–84%), yellow indicates slight undersupply (85–99%), and green indicates optimum coverage (100–120%) (data from the Department of Health—Abu Dhabi [11])

Recent estimates indicate that there is an imbalance in the supply of specialists and consultants both across the seven emirates and within each emirate [15]. In Abu Dhabi in 2015, there was an oversupply of some specializations such as general medicine and gynecology (i.e. government facilities), while there was a severe undersupply of physician specializing in trauma and injury, substance use (alcohol and other drugs), and oncology [29]. In 2016, there were no reported specialties with oversupply beyond demand; however, rheumatology, pediatric surgery, and trauma and injury are classified as areas in significant undersupply (0–60% coverage) [11]. The presented categories for the emirate level service line coverage, defined in the Capacity Master Plan of Department of Health Abu Dhabi, show the percentage of the available medical specialties over demand (Fig. 1). These categories slightly changed between 2015 and 2016 where any specialty was considered as oversupply (previously defined as > 130%).

One of the challenges of the UAE in terms of health workforce is the reliance on expatriate workers [3, 30, 31]. In Abu Dhabi, 82% of the population are non-nationals and 96% of the healthcare workers in the emirate are also expatriates from a wide range of countries. In Dubai, the expatriate population is 91% [32] and among healthcare workers 90% are expatriates (Table 1) [12]. The large proportion of expatriates working in the healthcare sector is a challenge to healthcare human resources as among the expatriate population there is a high turn-over of staff [3, 31] and a subsequent delay of replacing professionals especially in some specialties and sub-specialties. Hannawi and Salmi report that employment of healthcare workers in the UAE can be delayed due to the stringent licensing regulations required by different emirates and entities [31]. Expatriate health workers are recruited from a wide range of low-, middle-, and high-income developing and developed countries, which creates some heterogeneity in terms of training backgrounds, skills, and experience that may potentially affect patient-related outcomes, hospital performance, and/or overall health system performance. However, there is currently no publicly available data to support this notion. Nonetheless, increasing the number of UAE nationals qualified in medicine, nursing, and other allied health professionals, ideally trained within the UAE health system, will build a more stable health workforce and reduce the dependence on expatriates.

**Table 1:** Healthcare workers in Abu Dhabi per category and nationality (data from [3])

Profession	Abu Dhabi Emirate per 10 000 2015	Nationals 2015	Non-Nationals 2015	Total Abu Dhabi Emirate 2016	Abu Dhabi Region 2016	Al Ain Region 2016	Al Dhafra Region 2016
Physicians	52.4	10.5%	89.3%	8983	6325	2227	431
Dentists	*	8.8%	91.2%	1734	1214	472	48
Nurses and Midwives	134.7	0.7%	98.5%	24 915	17 663	6156	1096
Allied Health Professionals	*	6.0%	94.0%	7767	5855	1 604	308
Pharmacists	*	2.3%	97.6%	3348	2367	828	153

^*^There is no data on the number of these professionals per 10 000 population

Statistics shown in Table 1 highlights the need for further development of medical education and more specialist training for UAE healthcare professionals. [3] to decrease the imbalance of professionals across the regions of the Abu Dhabi emirate.

### The CCM gaps and the health workforce

A systematic review applied the CCM framework to identify the gaps in the primary healthcare clinics in Abu Dhabi’s health system [4]. In this present article, we used the identified gaps as a framework to propose specific recommendations for potential areas of improvement focusing on the healthcare workforce. The specific recommendations were a result of a search of the literature [6, 18, 31, 33, 34] on the topic, and the results are presented in Table 2. As advanced by the CCM, the reorganization towards chronic care services requires a set of new skills from health professionals. The use of technology to help healthcare workers become more efficient with completing the administrative tasks required by a health insurance system should provide sufficient time during the patient interaction for face-to-face communication. The appropriate use of information technology can provide additional contributions to performance, safety, and communication with patients [35]. Some of the listed recommendations are currently implemented in some, but not all, primary healthcare centers, named Ambulatory Healthcare System clinics.

**Table 2:** The use of the Chronic Care Model gaps as an example to develop healthcare workforce in the United Arab Emirates—Abu Dhabi

CCM elements	CCM gaps in primary health care services	Health workforce patient interaction issues
Health system	Promote effective improvement strategies aimed at comprehensive system change	• Apply *Plan-Do-Study-Act*.
	Visibly support coordination and improvement at all levels of the health system organization, beginning with the senior leadership team	• Provide some degree of independence to the healthcare team leaders (e.g., hospital/clinic directors).
Self-management support	Use effective self-management support strategies that include assessment, goal-setting, action planning, problem-solving, and follow-up	• Increase the number of nurse health educators to empower all patients to self-manage their chronic diseases. • Educate active community members to engage patients. • Disseminate health education materials through multiple channels (e.g., printed, electronic, social media, community initiatives.
Community	Encourage patients to participate in effective community programs	• Foster the development of community-based health programs, such as walking clubs for diabetes patients.
	Form partnerships with community organizations to support and develop interventions that fill gaps in needed services	• Harmonize the different entities dealing with chronic diseases to work together to promote patient well-being with their own disease, for example Emirates Diabetes Foundation, Imperial College of Diabetes, Universities.
	Advocate for policies to improve patient care	• Create healthcare worker stakeholder teams to ensure policy makers understand the opportunities and barriers at the implementation level.
Delivery system design	Define roles and distribute tasks among team members	• Improve leadership among the hierarchy chain within all the team members.
	Use planned interactions to support evidence-based care	• Create online scheduling reminders.
	Provide clinical case management services for complex patients	• Invest in clinical case managers who focus on communicating with the patient’s family and support services.
	Ensure regular follow-up by the care team	• Employ the family medicine doctor concept whereby they act as the gatekeeper to specialist services. • Allocate one primary healthcare, doctor or nurse, to each patient with regular appointments. • Develop a secure messaging communication system between patients and physicians/nurses.
Decision support	Embed evidence-based guidelines into daily clinical practice	• Promote the discussion of clinical guidelines among healthcare workers by allocating specific times/events for this purpose (e.g., bi-monthly journal club/seminar).
	Share evidence-based guidelines and information with patients to encourage their participation	• Foster teams to translate clinical guidelines into interactive materials for patients in their native language according to their level of health literacy. • Train health professionals to use information systems that fully integrate shared decision-making into the patient flow.
Clinical information system	Identify relevant subpopulations for proactive care	• Train clinical staff with digital knowledge and skills to be able to target high risk groups through electronic health records. • Train staff to extend their reach to patients with care gaps. • Incorporate the reach-out for patients with care gaps into the clinical information system.
	Provide timely reminders for providers and patients	• Design the clinical information systems to send reminder messages to patients for their appointments. • Develop and implement clinical information systems to notify doctors with patients that have examinations that are overdue or with guidelines according to the diagnosis.
	Share information with patients and providers to coordinate care	• Encourage people to use and connect with the health system through Mallafi—the Electronic Health Register brand.
	Monitor performance of practice team and care system.	• Develop live dashboard systems by individual and practice performances.

**Fig. 2:**
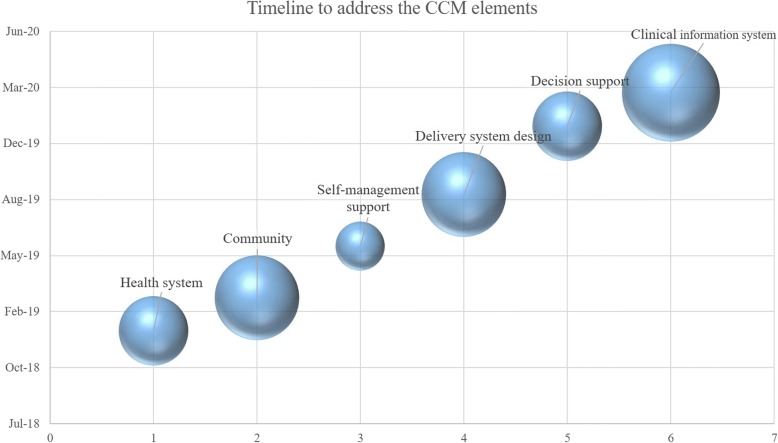
The elements of the CCM, by size of the gap (larger bubble diameter equates to a larger gap), and timeline to address them

A timeline has been developed as an example to improve each of the six elements of the CCM to help the Abu Dhabi emirate and ultimately the country to achieve the UAE National Vision 2021 of achieving world-class healthcare. In Fig. 2, the bubbles represent each element of the CCM. The size of the bubbles are according to the number of CCM element’s strategies that were not addressed, and they are displayed according to the timeline.

## Discussion

The population of licensed healthcare professionals in Abu Dhabi emirate increased by approximately 7000 (~ 17%) from 2015 to 2016. Despite these statistics, the emirate has an imbalance of healthcare workers within its regions and the country is still in shortage of trained health professionals, especially in some key specialties. Currently, the UAE has a young population structure, and the prevalence of chronic diseases is projected to increase over the next decade in both Abu Dhabi emirate and across the entire UAE due to population aging [4]. Health outcomes and the primary causes of mortality are similar across the seven UAE emirates; however, the numbers of nurses and physicians per 10 000 population in Abu Dhabi are higher than the UAE average, but lower when compared to Dubai [28, 36]. Overall, the UAE’s health system is quite unique as it comprises several healthcare regulators including healthcare free zones (Dubai and Sharjah) to encourage medical tourism and different providers from the federal and emirate level. In order to provide a comprehensive review of the UAE’s health workforce, a focused analysis on the healthcare workforce in the other emirates is needed. To address and eliminate inter-emirate disparities in the health workforce, the different health regulators in the UAE, with varying processes for licensing healthcare workers, should harmonize their processes to accelerate the employment of professionals, making the process easier for both employers and healthcare workers. For example, a nurse with a license to work in the emirate of Abu Dhabi needs to transfer their working license to the emirate of Dubai (which has its own healthcare regulator agency) and this process can be time-consuming and expensive. To address the high reliance on expatriate workers, the UAE may want to consider organizing a UAE-wide human resources for health planning exercise that includes prioritizing capacity building for UAE national healthcare professionals across all disciplines, specialties, and sub-specialties. The Department of Health Abu Dhabi has developed a recent Capacity Master Plan for Abu Dhabi, and this strategy includes some immediate measures to address the imbalance of specialists and consultants per specialty by evaluating the market prior to licensing new physicians, analyzing supply over demand [29]. There is no data available on the nationality of expatriate healthcare workers in Abu Dhabi, to compare if it is proportional to the nationalities of the population living in the emirate. However, it is known that the majority of the healthcare workforce are recruited from other Arab countries, South Asia (predominantly India), and South-East Asia (predominantly the Philippines) with a minority from Australasia, Europe, and North America [6]. In 2013, two other studies also identified additional challenges within the healthcare workforce including “low staff numbers, moral problems, skills imbalance, and the geographical misdistribution” [6, 31].

One of the strategies identified by our case study to enhance delivery system design was to invest in family medicine doctors; according to the new Department of Health Standard for Primary Care in Emirate of Abu Dhabi, primary care teams led by a family medicine physician are being implemented in clinics, centers, and hospitals [11]. The emirate of Abu Dhabi is moving towards the achievement of the Abu Dhabi Healthcare Strategic Plan, and one of the key measures implemented was the adoption of the PCMH by the Ambulatory Healthcare Services in 2013 [33] aligned with the CCM.

Continued investment in well-trained healthcare workers with strong leadership skills is required to improve the CCM gaps in the emirate of Abu Dhabi. In addition, further development and implementation of eHealth, such as SEHA’s eMallafi app, will facilitate the achievement of the Abu Dhabi Healthcare Strategic Plan. The correct implementation of eHealth into the healthcare organizations will require adjustments in the entire institution, including from the healthcare workforce [37]. More than simply increasing the number of healthcare workers in the needed areas, the aim is to optimize it, ensuring that they have the skills, competencies, and experience necessary to expand the UAE health system. This paper discusses additional specific areas that may require attention and investment to fulfill the UAE vision of achieving a World-Class Healthcare by 2021. As such, there needs to be an increased focus on the ratio, specialty, distribution, skill-mix, and performance of the health workforce in order to achieve the UAE National Vision 2021.

### Future considerations

The UAE population is expected to continue growing in size over the next decade due to natural growth and high inward migration of expatriates. However, the age distribution of the national population will change as total fertility rates decline and life expectancy increases. Consequently, the Abu Dhabi emirate and UAE will need to be prepared for this change in population size and structure and subsequent increased rates of chronic diseases in the adult population. Abu Dhabi is a good example of how health workforce management is being considered to address the challenge of delivering high-quality chronic care services to a significant proportion of the population. Healthcare services and workers need to be planned and prepared in advance to face these challenges and to achieve the criteria for a world-class healthcare by 2021. The CCM framework provides robust guidance to help align the new skill demands of the health workforce with the changing socio-demographic and health needs of both the Abu Dhabi and UAE population. Other high-income and/or rapidly developing countries with similar population growth and demographic changes may find this case study useful when developing future healthcare strategies and reforms.

## Abbreviations

CCM: Chronic Care Model
PCMH: Patient-Centered Medical Home
UAE: United Arab Emirates
WHO: World Health Organization
